# Influenza A H5N1 and HIV co-infection: case report

**DOI:** 10.1186/1471-2334-10-167

**Published:** 2010-06-14

**Authors:** Annette Fox, Peter Horby, Nguyen Hong Ha, Le Nguyen Minh Hoa, Nguyen Tien Lam, Cameron Simmons, Jeremy Farrar, Nguyen Van Kinh, Heiman Wertheim

**Affiliations:** 1 Oxford University Clinical Research Unit Viet Nam, Wellcome Trust Major Overseas Program, National Hospital of Tropical Diseases, 78 Giai Phong Road, Dong Da, Ha Noi, Viet Nam; 2 Centre for Tropical Medicine, Nuffield Department of Clinical Medicine, University of Oxford, Churchill Hospital, Old Road, Oxford OX3 7LJ, UK; 3 National Hospital of Tropical Diseases, 78 Giai Phong Road, Dong Da, Ha Noi, Viet Nam; 4 Oxford University Clinical Research Unit Viet Nam, Wellcome Trust Major Overseas Program, Hospital for Tropical Diseases, 190 Ben Ham Tu Street, District 5, Ho Chi Minh City, Viet Nam; 5 South East Asia Infectious Diseases Clinical Research Network, JI Diponegoro no 69, Jakarta, 10430, Indonesia

## Abstract

**Background:**

The role of adaptive immunity in severe influenza is poorly understood. The occurrence of influenza A/H5N1 in a patient with HIV provided a rare opportunity to investigate this.

**Case Presentation:**

A 30-year-old male was admitted on day 4 of influenza-like-illness with tachycardia, tachypnea, hypoxemia and bilateral pulmonary infiltrates. Influenza A/H5N1 and HIV tests were positive and the patient was treated with Oseltamivir and broad-spectrum antibiotics. Initially his condition improved coinciding with virus clearance by day 6. He clinically deteriorated as of day 10 with fever recrudescence and increasing neutrophil counts and died on day 16. His admission CD4 count was 100/μl and decreased until virus was cleared. CD8 T cells shifted to a CD27^+^CD28^- ^phenotype. Plasma chemokine and cytokine levels were similar to those found previously in fatal H5N1.

**Conclusions:**

The course of H5N1 infection was not notably different from other cases. Virus was cleared despite profound CD4 T cell depletion and aberrant CD8 T cell activation but this may have increased susceptibility to a fatal secondary infection.

## Background

Influenza A/H5N1 infection is characterized by high viral loads, overproduction of pro-inflammatory cytokines and chemokines, direct lung tissue destruction, pulmonary oedema and extensive inflammatory infiltration [1-3]. The prevailing view is that alveolar damage is the primary pathology leading to acute respiratory distress, multiple organ dysfunction syndrome and death [3]. Likewise, 2009 H1N1 infection can cause acute respiratory distress syndrome and death in previously healthy young adults very similar to the clinical syndrome seen in H5N1 [4].

It remains unclear whether lung pathology in severe influenza is a direct consequence of high viral loads and/or of ensuing inflammatory responses. The involvement of innate versus adaptive immunity in inflammation or controlling viremia is also poorly defined. Further understanding of the pathological processes is necessary to develop interventions that prevent severe lung disease. The occurrence of H5N1 infection in a patient with HIV infection offered a unique opportunity to study the pathological and immunological process when adaptive immunity is impaired.

### Case presentation

**Figure 1:**
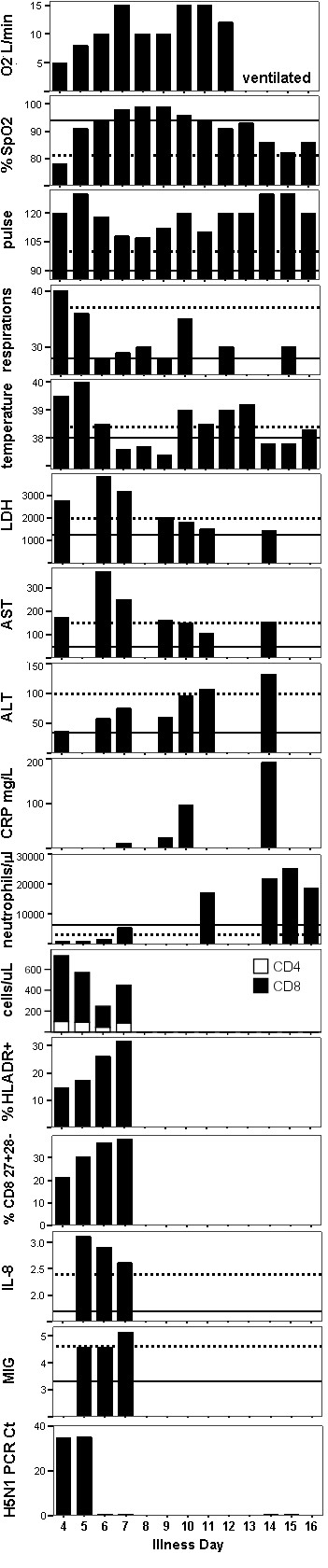
**Clinical and laboratory findings by day of illness**. Dashed and solid lines represent reported values for fatal and surviving H5N1 patients, respectively [2,8,9]. %HLADR+ is for the the CD8 T cell subset. Cytokines and chemokines are reported as Log 10 pg/ml.

In February 2009 a 30-year-old male was admitted to our hospital with a four-day history of fever, cough and increasing difficulty breathing. Three days prior to illness onset he had slaughtered, prepared and consumed a duck that was the last survivor of a household flock of ten birds, which had died over the preceding week. Close contacts did not report recent respiratory illness and the patient had no known chronic health conditions. On admission the patient was febrile, tachycardic, tachypneic and hypoxemic (Figure 1). Chest x-ray showed bilateral pulmonary infiltrates and an ultrasound revealed a right pleural-effusion. A throat swab was positive for influenza A/H5N1 by a real time RT-PCR protocol described elsewhere [2] but the cycle threshold (CT) value was 35 (Figure 1), indicative of low viral loads. Viral RNA was not detected in plasma. HIV antibody and/or antigen tests were performed as per routine practice in the admitting hospital. Determine^®^HIV-1/2 rapid test (Abbott Laboratories), Genscreen ULTRA HIV Ag-Ab (BioRad) and SFD HIV 1/2 (Fujirebio) tests were positive. HIV branched DNA load was 510 copies/ml (Quantiplex HIV RNA 2.0 Assay, Chiron Corporation, USA). The patient was commenced on supplemental oxygen by face mask, Oseltamivir phosphate (150 mg bd), broad-spectrum antibiotics (Table 1) for suspected bacterial co-infection and high dose co-trimoxazole for possible *Pneumocystis jiroveci *infection. Methylprednisolone was given 40 mg once a day from days 5 to 8 and 20 mg on days 9 and 10 of illness.

**Table 1:** Antibiotics and antifungals given

Drug name	Days of illness
Ceftazidine	4-10
Levofloxacin	4-14
Cotrimoxazole	5-16
Imipenem/Cilastatin	10-14
Fluconazole	10-14
Itraconazole	14-16
Cefperazone-sulbactam	14 -16

**Figure 2:**
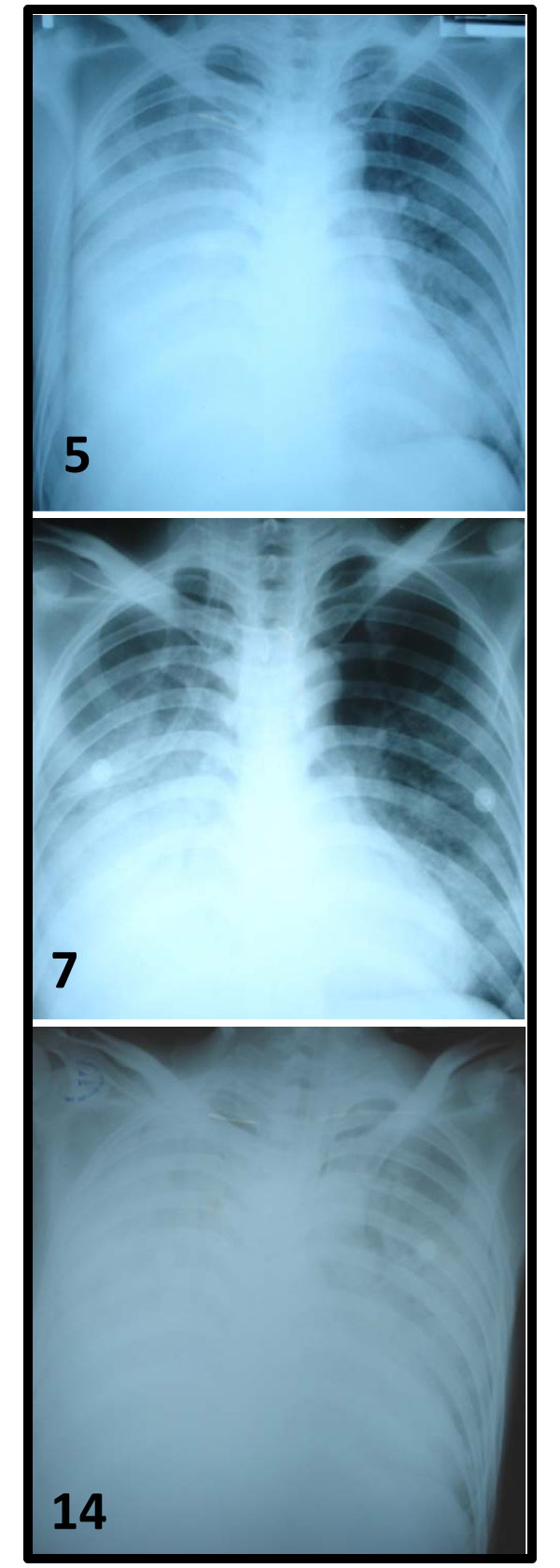
**Chest X-ray images**. The illness day is shown in the bottom left corner of each image.

At admission, clinical and laboratory signs were similar in severity to those reported previously for fatal H5N1 patients (Figure 1). The patient's condition improved over the next days coinciding with virus clearance but began to deteriorate again from day 10 of illness with a recrudescence of fever (Figure 1). Deterioration coincided with increasing neutrophil counts and CRP levels (Figure 1). Fluconazole was given from day 10 and the supplemental oxygen flow rate increased. Sputum and blood obtained on day 4 and 10 were assessed by smear and/or culture for bacteria and fungi but pathogenic organisms were not detected (Table 2). *Aspergillus fumigatus *was cultured from tracheal aspirate obtained on day 14, and fluconazole was replaced with itraconazole. Chest x-ray on day 14 showed marked bilateral infiltrates and pleural effusions (Figure 2). The patient was intubated and ventilated on day 15 of illness. The patient died on day 16 with respiratory and renal failure.

**Table 2:** Laboratory tests for co-infection

Illness Day	Specimen	Test	Result
4	blood	culture	negative after 5 days

4	sputum	smear and Ziehl-Neelsen stain	negative
		
		culture for bacteria and fungi	*Candida albicans*

4	N/A	Mantoux/PPD skin test	negative

10	blood	culture	negative after 5 days

10	sputum	culture for bacteria and fungi	*Candida albicans*

13	blood	culture	negative after 5 days

14	tracheal aspirate	smear for fungi	positive
		
		smear and Ziehl-Neelsen stain	negative
		
		culture for bacteria and fungi	*Candida albicans Aspergillus fumigatus*

Blood samples were available for immunological assessment until day 7 of illness. CD4 lymphopenia was marked (Figure 1) with a CD4 count of 100/μl and a CD4:CD8 ratio of 0.16 at admission, which is lower than previously reported for any H5N1 case [2]. CD4 and CD8 counts decreased until virus was cleared (Figure 1). The percentage of CD8 T cells expressing activation markers including HLA-DR (Figure 1) increased to reach levels at least 10 times higher than normal [5]. At admission 50% of CD8 T cells had a fully differentiated (CD27^-^CD28^-^) phenotype but this decreased with a concomitant increase in the percentage with an intermediate differentiation (CD27^+^CD28^-^) phenotype (Figure 1).

Chemokine and cytokine levels were measured using cytometric bead array kits (Becton Dickinson). MIG (CXCL9), IL-8 (Figure 1), IP-10 (CXCL10), MCP-1 (CCL2), IL-6 and IFN-γ (not shown) concentrations were increased to levels similar to those found previously in patients with fatal H5N1. IL-8, MCP-1 and IL-6 levels declined from day 5 to 7 whereas MIG and IP-10 increased.

## Conclusions

To our knowledge only one other patient with documented HIV and H5N1 co-infection has been reported and details of the clinical course in this patient have not been published [6].

Patients with immune compromise including those with HIV are at higher risk of complications associated with seasonal influenza [7]. This may not translate to highly pathogenic H5N1 which has been fatal in the majority of previously healthy patients. Although early clinical and laboratory findings were similar to those of other fatal H5N1 patients [2,6,8,9], the patient showed a transient clinical improvement and a relatively delayed time to death, which was accompanied by signs of secondary pneumonia. In addition, viral loads were relatively low with no virus detection in plasma, unlike some fatal cases described previously [2,6,8,9] suggesting that H5N1 viral clearance was not compromised by HIV co-infection. H5N1 can be cleared without antivirals and there is little benefit of Oseltamivir after day 6 of illness [9,10]. However early control of viral load appears to be important because survival rates are highest in patients treated earliest [10]. Thus, the contribution of Oseltamivir may have been negligible in this patient because viral loads were relatively low prior to administration. Lymphopenia is common in H5N1 patients [2,6,9] and low CD3 counts and CD4:CD8 ratios have been reported [2]. We can not determine if immune compromise preceded influenza A/H5N1 infection in this patient. Investigation of AIDS defining illnesses was not exhaustive because the patient was reported to have been healthy and the presenting symptoms and rapid onset of illness were consistent with the confirmed diagnosis of H5N1. We can not exclude the possibility of *P. jirovecci *or mycobacterial co-infection. CD4 counts and CD4:CD8 ratios were lower than we have seen in other H5N1 patients (unpublished findings). This suggests that CD4 T cells may not be required for viral clearance consistent with findings that CD8 T cells are more important for survival from highly virulent influenza infection in mice [11]. The patient maintained substantial numbers of peripheral CD8 T cells and a large fraction became activated, but the concomitant shift to a CD27+CD28- phenotype raises doubts about their antiviral function. This phenotype is associated with CD8 T cells that proliferate but lack cytotoxic function and accumulate in progressive HIV infection [12]. Chemokine and cytokine levels were high as in other fatal H5N1 cases and may account for the severity of the early pneumonia. The prevailing view is that excessive cytokine and chemokine release are secondary to high viral loads [2], whereas viral loads were low in this patient. Although HIV infection is also associated with increased expression of proinflammatory cytokines [13,14], levels tend to be lower than found here [15], even with co-presentation of pneumocystis pneumonia, bacterial pneumonia or mycobacteriosis, and IFN-γ expression is often decreased [13]. Levels of IL-8, MCP-1, and IL-6 also decreased with H5N1 viral load indicting that they are primarily induced by H5N1 infection. There has been no conclusive report of secondary infection accompanying H5N1 whereas secondary pneumonia may have caused a considerable fraction of the deaths from the highly pathogenic 1918 H1N1 strain and the current H1N1 2009 strain [16]. Findings in this patient including recrudescence of fever, rising CRP levels and neutrophilia were consistent with a secondary infection despite being treated with broad-spectrum antibiotics. *A. fumigatus *was detected in a tracheal aspirate suggestive of invasive pulmonary aspergillosis. Corticosteroid adminsitration may have contributed to the presumed development of invasive pulmonary aspergillosis [17], but there have been numerous reports of invasive pulmonary aspergillosis developing after influenza A infection in which T cell lymphopenia occurred [18,19]. None these patients had HIV and few received corticosteroids indicating the influenza A associated immune suppression may be sufficient for the development of invasive pulmonary aspergillosis. In conclusion the course of H5N1 infection in this case was not notably different in the presence of HIV co-infection but it is possible that HIV co-infection and profound CD4 T cell depletion increase susceptibility to secondary infection. The findings suggest that CD4 T cells may not be required for H5N1 virus clearance.

### Consent

The admitting hospital approved the use of patient samples and data and written informed consent was obtained from the next of kin for publication of this case report. A copy of the written consent is available for review by the Editor-in-Chief of this journal.

## Competing interests

The authors declare that they have no competing interests.

## Authors' contributions

AF designed and conducted immunological assessments and drafted the manuscript; PH coordinated the collection of clinical information and helped draft the manuscript; NHH and NTL were the reference physicians during the in-hospital management of this case; LNMH carried out molecular diagnostics and immunological assessments; CS and JF participated in study design and analysis; NVK helped coordinate the collection of patient information; HW coordinated virology and helped draft the manuscript. All authors read and approved the final manuscript.

## Pre-publication history

The pre-publication history for this paper can be accessed here:

http://www.biomedcentral.com/1471-2334/10/167/prepub
